# The Use and Abuse of Tobacco

**Published:** 1868-02

**Authors:** B. C. Brodie


### SELECTED ARTICLES.

---

### ARTICLE V.

### *The Use and Abuse of Tobacco.*

[We would call especial attention to the following letter from Sir Benjamin Brodie. The long and extensive experience and mature judgment of the writer entitle his opinions to the greatest weight.]

"Sir: Having been applied to some time since to join in a petition to the House of Commons, that they would appoint a committee to inquire into the effects produced by the prevailing habit of tobacco smoking. I declined to do so; first, because it did not appear to me that such a committee would be very competent to discuss a question of this kind; and, secondly, even if they were so, I

did not see that it would be possible for Parliament to follow up by any act of legislation the conclusions at which they might have arrived. Nevertheless I am ready to admit that the subject is one of no trifling importance, and well worthy the serious consideration of any one who takes an interest in the present and future well-being of society. From these considerations it is that I now venture to address you the following observations.

"The empyreumatic oil of tobacco is produced by distillation of that herb at a temperature above that of boiling water. One or two drops of this oil (according to the size of the animal) placed on the tongue will kill a cat in the course of a few minutes. A certain quantity of the oil must be always circulating in the blood of an habitual smoker, and we cannot suppose that the effects of it upon the system can be merely negative. Still, I am not prepared to subscribe to the opinion of those who hold that, under all circumstances, and to however moderate, an extent it be practiced, the smoking of tobacco is prejudicial. The first effect of it is to soothe and tranquilize the nervous system. It allays the pains of hunger, and relieves the uneasy feelings produced by mental and bodily exhaustion. To the soldier who has passed the night in the trenches before a beleaguered town, with only a distant prospect of breakfast when the morning has arrived; to the sailor, contending with the elements in a storm; to the traveller in an uncultivated region, with an insufficient supply of food, the use of a cigar or a tobacco pipe may not only be a grateful indulgence, but really beneficial. But the occasional use of it under such circumstances is a very different matter from the habit of constant smoking which prevails in certain classes of society at the present day.

"The effects of this habit are, indeed, various, the difference depending on difference of constitution, and difference in the mode of life otherwise. But, from the best observations which I have been able to make on the subject, I am led to believe that there are very few who do not suffer harm from it, to a greater or less extent. The earliest symptoms of it are manifest in the derangement of the nervous system. A large proportion of habitual smokers are rendered lazy and listless, indisposed to bodily and incapable of much mental exertion. Others suffer from depression of the spirits, amounting to hypochondriasis, whi^h smoking relieves for a time, though it aggravates afterwards. Occasionally there is a general nervous excitability, which, though very much less in degree, partakes of the nature of the *delirium tremens* of drunkards. I have known many individuals to suffer from severe nervous pains, sometimes in one, sometimes in another part of the body. Almost the worse case of neuralgia that ever came under my observation was that of a gentleman who consulted the late Dr. Bright and myself. The pains were universal and never absent; but during the night they were especially intense, so as almost wholly to prevent sleep. Neither the patient himself nor his medical attendant had any doubts that the disease was to be attributed to his former habit of smoking, on the discontinuance of which he slowly and gradually recovered. An eminent surgeon, who has a great experience in ophthalmic diseases, believes that, in some instances, he has been able to trace blindness from amaurosis to excess in tobacco smoking; the connection of the two being pretty well established in one case by the fact that, on the practice being left off, the sight of the patient was gradually restored. It would be easy for me to refer to other symptoms indicating deficient power of the nervous system to which smokers are liable; but it is unnecessary for me to do so; and, indeed, there are some which I would rather leave them to imagine for themselves than undertake the description of them myself in writing.

"But the ill effects of tobacco are not confined to the nervous system. In many instances there is a loss of the healthy appetite for food, the imperfect state of the digestion being soon rendered manifest by the loss of flesh and the sallow countenance. It is difficult to say what other diseases may not follow the imperfect assimilation of food continued during a long period of time. So many causes are in operation in the human body which may tend in a greater or less degree to the production of organic changes in it, that it is only in some instances we can venture to pronounce as to the precise manner in which a disease that proves mortal has originated. From cases, however, which have fallen under my observation, and from a consideration of all the circumstances, I cannot entertain a doubt that, if we could obtain accurate statistics on the subject, we should find that the value of life in habitual smokers is considerably below the average. Nor is this opinion in any degree contradicted by the fact that there are individuals who in spite of the inhalation of tobacco smoke live to be old, and without any material derangement of the health; analogous exceptions to the general rule being met with in the case of those who have indulged too freely in the use of spirituous and fermented liquors.

"In the early part of the present century tobacco smoking was almost wholely confined to what are commonly called the lower grades of society. It was only every now and then that any one who wished to be considered as a gentleman was addicted to it. But since the war on the Spanish Peninsula, and the consequent substitution of the cigar for the tobacco-pipe, the case has been entirely altered. The greatest smokers at the present time are to be found, not among those who live by their bodily labor, but among those who are more advantageously situated, who have better opportunities of education, and of whom we have a right to expect that they should constitute the most intelligent and thoughtful members of the community. Nor is the practice confined to grown-up men. Boys, even at the best schools, get the habit of smoking, because they think it manly and fashionable to do so; not unfrequently because they have the example set them by their tutors, and partly because there is no friendly voice to warn them as to the special ill consequences to which it may give rise where the process of growth is not yet completed, and the organs are not yet fully developed.

"The foregoing observations relate to the habit of smoking as it exists among us at the present time. But a still graver question remains to be considered. What will be the result if this habit be continued by future generations? It is but too true that the sins of the fathers are visited upon their children and their children's children. We may here take warning from the fate of the red Indians of America. An intelligent American physician gives the following explanation of the gradual extinction of this remarkable people: One generation of them became addicted to the use of fire-water. They have a degenerate and comparatively imbecile progeny, who indulge in the same vicious habit with their parents. Their progeny is still more degenerate, and after a very few generations the race ceases altogether. We may also take warning from the history of another nation, who some few centuries ago, while following the banners of Solyman the Magnificent, were the terror of Christendom, but who since then, having become more addicted to tobacco smoking than any of the European nations, are now the lazy and indolent Turks, held in contempt by all civilized communities.

"In thus placing together the consequences of intemperance in the use of alcohol and that in the use of tobacco, I should be sorry to be misunderstood as regarding these two kinds of intemperance to be in an equal degree pernicious and degrading.

"The inveterate tobacco smoker may be stupid and lazy, and the habit to which he is addictad may gradually tend to shorten his life and deteriorate his offspring, but the dram drinker is quarrelsome, mischievous, and often criminal. It is under the influence of gin that the burglar and the murderer become fitted for the task which they have undertaken. The best thing that can be said for dram-drinking, is that it induces disease, which carries the poor wretch prematurely to the grave, and rids the world of the nuisance. But, unfortunately, in this, as in many other cases, what is wanting in quality is made up in quantity. There are checks on one of these evil habits which there are not on the other. The dram-drinker, or, to use a more general term, the drunkard, is held to be a noxious animal. He is an outcast from all decent society, while there is no such exclusion for the most assiduous smoker.

"The comparison of the effects of tobacco with those of alcohol leads to the consideration of a much wider question than that with which I set out. In all ages of which we have any record, mankind have been in the habit of resorting to the use of certain vegetable productions, not as contributing to nourishment, but on account of their having some peculiar influence as stimulants or sedatives (or in some other way) on the nervous system. Tobacco, alcohol, the Indian hemp, the kava of the South Sea Islanders, the Paraguay tea, coffee, and even tea, being to this category. A disposition so universal may almost be regarded as an instinct, and there is sufficient reason to believe that, within certain limits, the indulgence of the instinct is useful. But we must not abuse our instincts. This is one of the most important rules which man, as a responsible being, both for his own sake and for that of others, is bound to observe. Even such moderate agents as tea and coffee, taken in excess, are prejudicial. How much more so are tobacco and alcohol, tending, as they do, not only to the degradation of the individual, but that of future generations of our species.

"If tobacco-smokers would limit themselves to the occasional indulgence of their appetite, they would do little harm either to themselves or others; but there is always danger that a sensual habit once begun may be carried to excess, and that danger is never so great as in the case of those who are not compelled by the necessities of their situation to be actually employed. For such persons the prudent course is to abstain from smoking altogether.

"Trusting that you and your readers will excuse me for occupied so large a space in your coulums,

"I am Sir, your humble servant,

"Aug. 17. B. C. Brodie."

—(*Med. Times and Gaz.*)

---

